# Two *FT*-like genes of the PEBP family constitute critical regulators of floral transition in *Prunella vulgaris*

**DOI:** 10.3389/fpls.2026.1953973

**Published:** 2026-10-06

**Authors:** Jingtian Liu, Jiayan Zhao, Xiaohan Wang, Jiayin Liu, Tong Xiao, Jun Zhu, Hua Yuan, Kunpeng Liu

**Affiliations:** 1 Shanghai Key Laboratory of Plant Functional Genomics and Resources, Shanghai Chenshan Plant Science Research Center, Chinese Academy of Science, Shanghai Chenshan Botanical Garden, Shanghai, China; 2 College of Life Sciences, Shanghai Normal University, Shanghai, China

**Keywords:** floral transition, genome-wide, *P. vulgaris* L., phosphatidylethanolamine-binding protein (PEBP) family, *PvuFT*

## Abstract

The primary medicinal tissues of *Prunella vulgaris* (Lamiaceae) are its spikes. Elucidating the molecular genetic determinants governing flowering time in *P. vulgaris* is therefore critical for ensuring the stable production of this herbal resource. Flowering is regulated by multiple exogenous and endogenous signals that ultimately converge on the floral integrator encoded by *FLOWERING LOCUS T* (*FT*). However, the regulatory network underlying flowering control in *P. vulgaris* remains largely unexplored. In this study, distinct flowering phenotypes were observed among four geographically distinct populations of *P. vulgaris*. To dissect the function of FT homologs in this process, we identified a total of 15 genes encoding phosphatidylethanolamine-binding protein (PEBP) within the *P. vulgaris* genome. Phylogenetic reconstruction coupled with duplication analysis revealed that gene duplication events have given rise to six *FT*-like paralogs. Subcellular localization assays showed that all six FT-like proteins are dually targeted to the nucleus and the peripheral cytoplasm. Among these, *PvuFT1* and *PvuFT2* exhibited predominant transcript accumulation in leaves, and their respective heterologous overexpression in *Arabidopsis thaliana* significantly accelerated floral transition. Furthermore, functional interrogation via a virus-induced gene silencing (VIGS) system, employed to knockdown *PvuFT1* and/or *PvuFT2* expression in *P. vulgaris* leaves, demonstrated that these two genes promote flowering in the native host. Collectively, our findings provide compelling evidence for the functional diversification of *FT*-like genes in the regulation of flowering time and offer a valuable genomic resource for marker-assisted breeding strategies in *P. vulgaris.*

## Introduction

*Prunella vulgaris* L. (‘Xia Ku Cao’ in Chinese) is a perennial herbaceous species belonging to the family Lamiaceae, with a native distribution spanning temperate regions of Asia and Europe (Liu et al., 2024). The dried spikes of *P. vulgaris*, traditionally harvested in early summer, have been employed in Chinese herbal medicine for over a century, primarily as a single agent or in combination with other botanicals, owing to their favorable efficacy profile and minimal adverse effects (Liu et al., 2025). Documented therapeutic applications include the alleviation of acute mastitis (Luo et al., 2024), the amelioration of sleep disturbances (Sayson et al., 2024), and the adjunctive management of hyperthyroidism (Zhang et al., 2024b). Beyond its pharmaceutical uses, *P. vulgaris* serves as a major ingredient in functional herbal teas. The annual domestic demand for its dried spikes in China having surpassed 5 million kilograms (Chen et al., 2019). Notably, the medicinally active spikes are reproductive structures that develop exclusively upon the initiation of flowering. Consequently, the precise modulation of flowering time represents a pivotal strategy for optimizing both the quality and the yield of this economically important medicinal herb. Although the molecular regulatory networks governing flowering time have been extensively characterized in model plant species (Montez et al., 2026), such mechanisms remain largely unexplored within the Lamiaceae family.

Flowering signifies a pivotal developmental shift from vegetative growth to reproductive competence in angiosperm (Zhao et al., 2005). To maximize environment adaptation and reproductive fitness, the timing of this transition is coordinately governed by multiple interconnected signaling cascades, including the age, photoperiod, autonomous, vernalization, light-quality, and hormonal pathways (Maple et al., 2024; Montez et al., 2026). These distinct routes converge upon a suite of central regulators collectively designated as “floral pathway integrators”, among which the most extensively characterized is florigen-a systemic signal encoded by *FLOWERING LOCUS T* (*FT*), a member of the PEBP family (Liu et al., 2016).

In *Arabidopsis thaliana*, the PEBP gene family is phylogenetically partitioned into four discrete subclades (Bellinazzo et al., 2024): the *FLOWERING LOCUS T* (*FT*)-like subgroup, comprising *AtFT* and its paralog *AtTSF*, is primarily associated with the production of florigenic signals (Turck et al., 2008; Yamaguchi et al., 2005). In contrast, the *TERMINAL FLOWER 1* (*TFL1*)-like clade, which includes *AtTFL1*, *AtATC*, and *AtBFT* functions predominantly as a floral repressor (Shannon and Meeks-Wagner, 1991; Kobayashi et al., 1999; Yoo et al., 2010; Mimida et al., 2001). The *MOTHER OF FT AND TFL1* (*MFT*)-like group, represented solely by *AtMFT*, participates in both flowering regulation and seed germination (Yoo et al., 2004; Xi and Yu, 2014), whereas the recently define *STEPMOTHER OF FT AND TFL1* (*SMFT*)-like clade, consisting of *AtSMFT*, appears to be specifically involved in germination control (Bellinazzo et al., 2024).

Although core flowering regulators are phylogenetically conserved across plants, the copy number and functional attributes of *FT*-like genes exhibit considerable interspecific divergence (Wickland and Hanzawa, 2015). Illustrative cases include sugar beet (*Beta vulgaris*), wherein two FT homologs-BvFT1 and BvFT2-execute opposing regulatory functions, with the Y134 and W138 residues within segment B serving as critical determinants of this function switch (Pin et al., 2010). In lychee (*Litchi chinensis*), natural polymorphisms within the promoter region of *LcFT1* modulate the binding affinity of the SVP protein, thereby altering *LcFT1* transcriptional output and consequently contributing to phenotypic variation in flowering time (Huang et al., 2024). Collectively, the systematic identification and functional dissection of PEBP family members hold substantial promise for advancing our understanding of the molecular frameworks underpinning flowering regulation and for informing the molecular breeding of economically important crops.

*P. vulgaris* is widely distributed across the southern regions of Qinling Mountains in China. The recent availability of a chromosome-scale genome assembly for *P. vulgaris* has now enabled systematic investigation into the genetic determinants of its floral transition (Zhang et al., 2024a; Liu et al., 2025). In this study, we observed pronounced inter-population differences in flowering time among four geographically distinct *P. vulgaris* accessions. Through comprehensive genome-wide identification of PEBP family members and subsequent functional characterization of FT-like homologs, we uncovered that *PvuFT1* and *PvuFT2* function as critical positive regulators of flowering in this species. Collectively, our findings establish that the modulation of *PvuFT1* and *PvuFT2* transcript abundance constitutes a key adaptive mechanism underlying flowering-time plasticity in *P. vulgaris*. These insights not only advance our understanding of floral regulation in non-model medicinal plants but also hold practical implications for optimizing the harvest schedule and supply chain management of *P. vulgaris* as a valuable pharmaceutical resource.

## Materials and methods

### Plant materials and growth conditions

Plant materials of *P. vulgaris* were collected from four geographically distinct localities-Guiyang, Liangping, Queshan, and Yiyang-and were planted in September 2024 in the experimental fields of Shanghai Chenshan Botanical Garden, with flowering phenology subsequently recorded in May 2025. For molecular characterization, *P. vulgaris* plants were cultivated in a growth chamber under long-day (LD) photoperiod conditions (16 h light: 8 h dark; 22 °C: 22 °C, light: dark). *Arabidopsis thaliana* plants used in this study were of the Col-0 ecotype and were grown either on half-strength Murashige and Skoog (MS) medium supplemented with 0.8% (w/v) sucrose or on soil, both under LD conditions (16 h light: 8 h dark; 22 °C: 22 °C, light: dark), following the protocol described previously (Liu and Sun, 2021).

### Identification of PEBP genes in *P. vulgaris*

To systematically identify PEBP family member, the hidden Markov model (HMM) profile PF01161, corresponding to the conserved PEBP domain, was retrieved from the Pfam database (https://www.ebi.ac.uk/interpro/entry/pfam/) using “PEBP” as the query term. This profile was subsequently employed to perform HMMER searches against the genome assemblies of *A. thaliana* and *P. vulgaris* genomes, respectively, to obtain the gene sequences of the PEBP families in *A. thaliana* and *P. vulgaris*, enabling the retrieval of candidate PEBP-encoding gene sequences (Garcia-Hernandez et al., 2002). Redundant transcripts sharing identical sequence identifiers were removed to retain a non-redundant set. The presence of the characteristic PEBP domain in each candidate was further validated using the NCBI Conserved Domains database (https://www.ncbi.nlm.nih.gov/cdd), thereby yielding the final repertoire of PEBP family genes in both species. For the *P. vulgaris* PEBP members, physicochemical properties-including molecular weight (MW), isoelectric point (pI), and grand average of hydropathicity (GRAVY)-were predicted using the ExPASy ProtParam online tool (https://web.expasy.org/protparam/) (Gasteiger et al., 1999). Subcellular localization was inferred via TargetP-2.0 (available at https://services.healthtech.dtu.dk/services/TargetP-2.0/), employing the default parameters for plant protein prediction.

### Phylogenetic analyses and duplication analysis

For phylogenetic reconstruction, multiple sequence alignments of the PEBP protein sequences from *P. vulgaris* and *A. thaliana* were performed using MEGA 10. A phylogenetic tree was subsequently constructed via the neighbor-joining (NJ) method, with nodal support assessed by 1000 bootstrap replicates. The resulting tree was visualized and annotated using the interactive online platform iTOL (https://itol.embl.de/). For gene duplication and synteny analyses, the *P. vulgaris* genome assembly and its corresponding GFF annotation file were imported into TBtools. Chromosomal localization information for all PEBP family members was extracted, and a genome-wide chromosome distribution map was generated. Inter- and intra-chromosomal collinearity relationship among PEBP-related loci were further investigated using the MCScanX-superfast module and the Advanced Circos functionality embedded within TBtools, with the resulting syntenic patterns visualized accordingly (Chen et al., 2023).

### Gene structure and conserved domain analysis

To characterize the structural organization of PEBP family members, exon–intron architectures were analyzed using SnapGene, based on the alignment of genomic nucleotide sequences with their corresponding coding sequences (CDSs). For conserved motif discovery, the protein sequences of all PEBP-related genes were submitted to the MEME Suite (https://meme-suite.org/meme/tools/meme), with the maximum number of motifs to be detected set to 10. The resulting predictions regarding motif composition and positional distribution were subsequently retrieved for comparative analysis (Bailey et al., 2009).

### Subcellular localization of PEBP protein

For subcellular localization analysis, the full-length coding sequences (CDSs) of *PvuFT1* through *PvuFT6*, along with *AtFT*, were amplified using gene-specific primers (**Supplementary Table 2**) and individually cloned into the binary vector pC131-N1-YFP (Liu et al., 2024), generating the fusion constructs *PvuFT1*-*YFP* through *PvuFT6*-*YFP*, as well as *AtFT*-*YFP*. The resulting recombinant plasmids, together with the empty YFP vector, were separately introduced into Agrobacterium tumefaciens strain GV3101 via transformation. Transformed agrobacterial cells were harvested, resuspended in infiltration buffer containing 10 mM MgCl_2_, 10 mM MES (pH 5.6), and 200 μM acetosyringone (AS), and adjusted to a final optical density (OD_600_) of 0.5. The cultures were then infiltrated into the abaxial surface of *Nicotiana benthamiana* leaves for transient expression. At 48–72 hours post-infiltration, yellow fluorescent protein (YFP) signals were examined using an Olympus FV10i laser scanning confocal microscope to determine the subcellular distribution of the fusion proteins.

### Plant transformation and phenotypic observation of transgenic *Arabidopsis*

The overexpression constructs-*PvuFT1*-*YFP* through *PvuFT6*-*YFP*, along with *AtFT*-*YFP*-were separately introduced into *Agrobacterium tumefaciens* strain GV3101 and subsequently delivered into *Arabidopsis thaliana* (Col-0) via the floral-dip method (Clough and Bent, 1998). Transgenic progeny was selected on 1/2 MS solid medium supplemented with 25 mg/L hygromycin. T_2_ transgenic lines derived from independent transformation events were employed for subsequent flowering-time phenotyping.

### RNA extraction and real-time quantitative PCR analysis

*P. vulgaris* accessions originating from Guiyang and Yiyang were selected as experimental materials. Leaf tissues were harvested at five time points-5, 6, 7, 8, and 9 weeks post-planting-with three independent biological replicates per time point. All collected samples were immediately flash-frozen in liquid nitrogen and subsequently stored at −80 °C until RNA extraction. Total RNA was isolated using a Plant RNA Extraction Kit, and first-strand cDNA was synthesized via reverse transcription with a commercially available cDNA synthesis kit. Quantification real-time PCR (qRT-PCR) was performed using the SYBR Green fluorescent quantification system. *Pvu06G020000* and *Actin2* were employed as internal reference genes for normalization. Relative transcript abundances were calculated using the 2^−ΔΔCt method (Rao et al., 2013).

### Virus-induced gene silencing

To suppress the expression of *PvuFT1* and *PvuFT2*, gene-specific coding sequences of each target gene were respectively amplified and cloned into the pTRV2 vector, generating the silencing constructs pTRV2-*PvuFT1* and pTRV2-*PvuFT2*. The resulting recombinant plasmids, along with the empty pTRV1 and pTRV2 vectors, were separately introduced into Agrobacterium tumefaciens strain GV3101. Agrobacterial cultures at the exponential growth phase were collected, resuspended in infiltration buffer containing 10 mM MgCl_2_, 10 mM MES (pH 5.6), and 200 μM acetosyringone, and adjusted to a final optical density (OD_600_) of 0.5. Equal volumes of the pTRV1- and pTRV2-derived suspensions were mixed thoroughly and then infiltrated into the abaxial surface of leaves from 5-week-old *P. vulgaris* at seedlings stage using a needleless syringe. Following infiltration, the inoculated plants were kept in darkness for 24 hours and subsequently transferred to normal growth conditions. The flowering time was recorded, and the transcript levels of *PvuFT1* and *PvuFT2* in the infiltrated leaves were examined via qRT-PCR to verify the silencing efficiency.

## Results

### *P. vulgaris* accessions from distinct geographical origins exhibit marked variation in flowering time

Flowering time of plants is generally considered to be modulated by both genetic determinants and environmental cues. To investigate the regulatory basis of flowering-time variation in *P. vulgaris*, we collected the germplasm accessions of *P. vulgaris* from four geographically disparate regions across China: Guiyang (Guizhou), Liangping (Chongqing), Queshan (Henan), and Yiyang (Jiangxi) (**Figure 1A**). These accessions were planted in September 2024 in the experimental field at Shanghai Chenshan Botanical Garden, and their flowering time was recorded in May 2025. As shown in **Figure 1B**, the Guiyang accession flowered earliest, followed sequentially by those from Liangping, Queshan, and Yiyang, with the latter exhibiting the latest flowering time. This inter-accession flowering-time hierarchy was consistently reproduced across two consecutive growing seasons (**Figure 1C**). Given that all accessions were cultivated under uniform environmental conditions, the observed divergence in flowering time strongly suggests a genetic basis underlying the regulation of floral transition in *P. vulgaris*.

**Figure 1:**
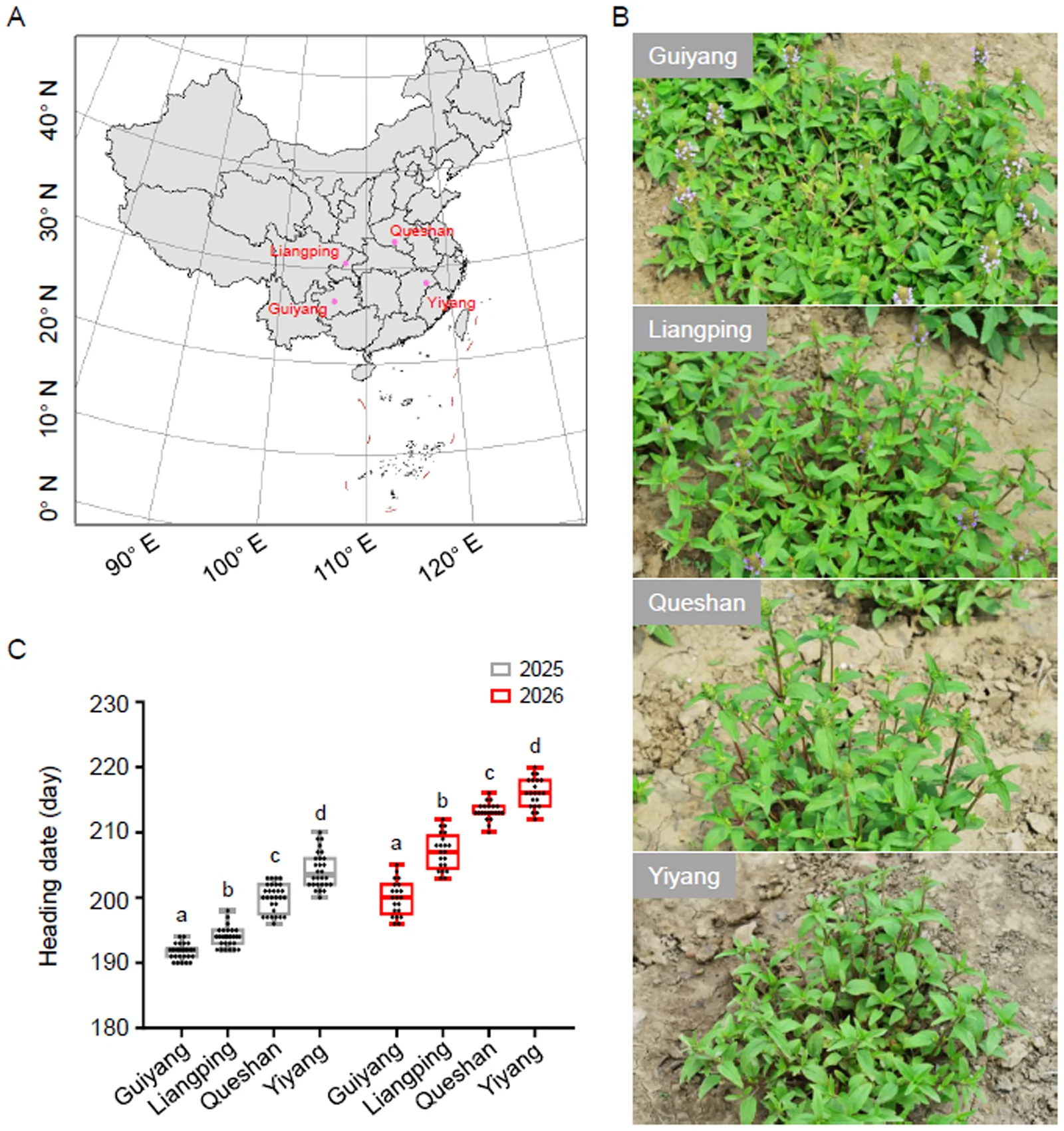
Flowering time variation among *P. vulgaris* ecotypes. **(A)** Geographic origins of the *P. vulgaris* populations sampled from Guiyang (Guizhou Province), Liangping (Chongqing Municipality), Queshan (Henan Province), and Yiyang (Jiangxi Provinces). **(B)** Representative phenotypes of the four populations cultivated under uniform field conditions at Shanghai Chenshan Botanical Garden. **(C)** Heading dates recorded for the four populations grown at Shanghai Chenshan Botanical Garden. Values represent the mean ± SD. Different lowercase letters denote statistically significant differences at *P* < 0.05, as determined by one-way ANOVA followed by Tukey’s *post-hoc* multiple comparison test.

### Genome-wide identification and characterization of PEBP family members in *P. vulgaris*

*FLOWERING LOCUS T* (*FT*), a member of the phosphatidylethanolamine-binding protein (PEBP) family, functions as a pivotal floral pathway integrator and plays a central role in the regulation of flowering time. To explore the potential contribution of *FT* homologs to flowering-time control in *P. vulgaris*, we performed a genome-wide hidden Markov model (HMM)-based search to identify PEBP family proteins in *P. vulgaris*. This approach led to the retrieval of 15 PEBP family members (**Supplementary Table 1**), which were subsequently designated according to their sequence similarity to annotated *Arabidopsis* homologs (**Figure 2A**). The identified gene set comprises *PvuFT1* through *PvuFT6*, *PvuBFT1*-*PvuBFT2*, *PvuATC1*-*PvuATC2*, *PvuTFL1A*-*PvuTFL1C*, *PvuMFT*, and *PvuSMFT* (**Figure 2A**). As summarized in **Table 1**, these 15 PEBP proteins exhibit considerable variation in their physicochemical properties. Their protein lengths range from 168 amino acids (PvuSMFT) to 226 amino acids (PvuTFL1B), which corresponding molecular weights spanning 18.29 kDa (PvuSMFT) to 25.10 kDa (PvuTFL1B) and isoelectric points ranging from 5.44 (PvuSMFT) to 9.60 (PvuMFT). All members display grand average of hydropathicity (GRAVY) values, indicative of their hydrophobic character. Subcellular localization predictions further revealed that all PEBP proteins are predominantly targeted to the nucleus and cytoplasm.

**Figure 2:**
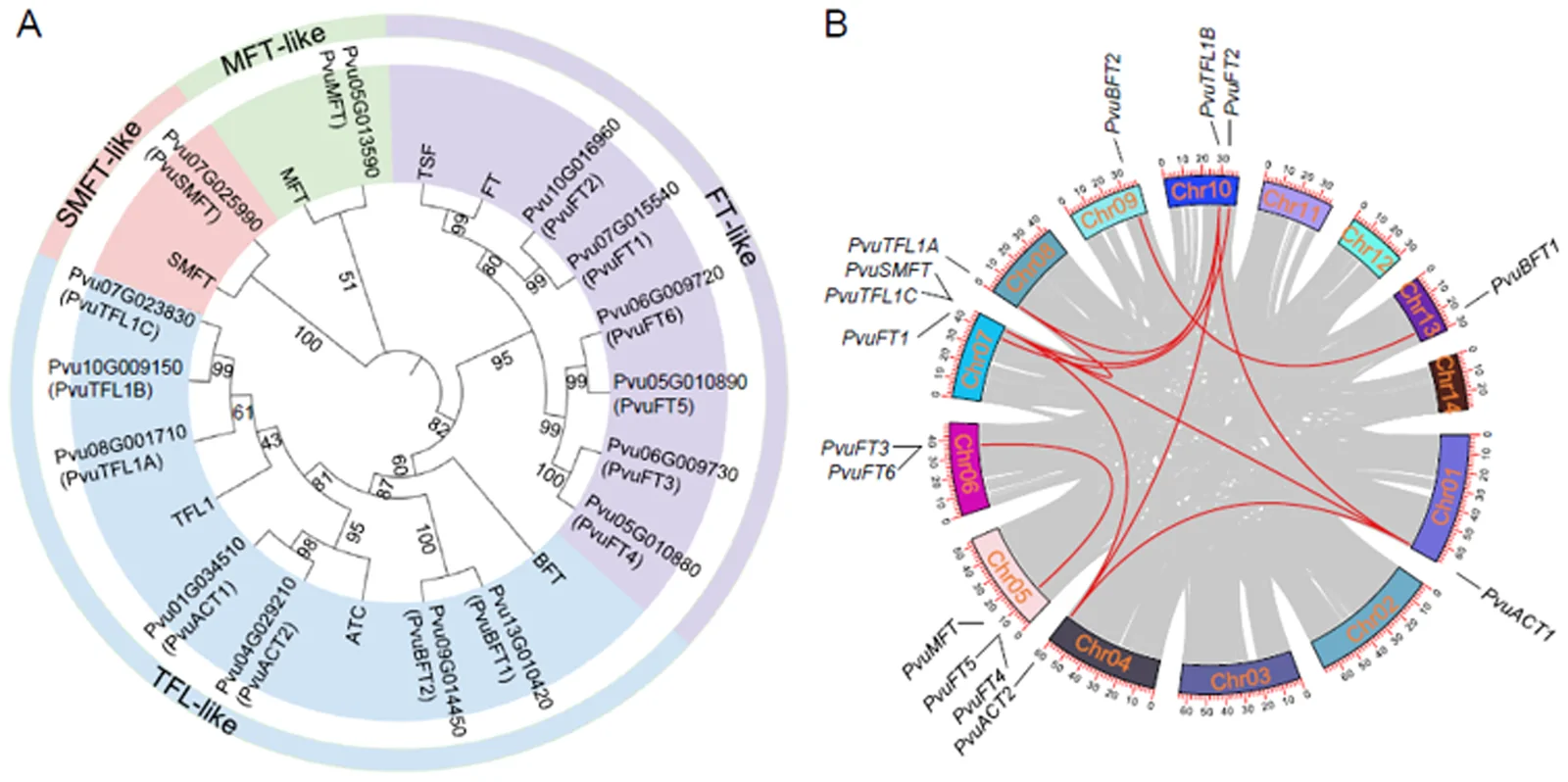
Phylogenetic and synteny analyses of PEBP family proteins in *P. vulgaris*. **(A)** Phylogenetic relationships of PEBP proteins from *P. vulgaris* and *A. thaliana*. The phylogenetic tree was constructed using the Neighbor-Joining (NJ) method implemented in MEGA 10 software. **(B)** Duplication and synteny analysis of the PEBP gene family in *P. vulgaris.* Homologous gene pairs are denoted by red lines.

**Table 1:** PEBP protein family physicochemical characterization in *P. vulgaris*.

Gene	Gene ID	Protein/ AA	MW (kDa)	Pl	GRAVY	Subcellular localization
*PvuFT1*	Pvu07G015540	176	19.94	8.71	-0.385	Nucleus, Cytoplasm
*PvuFT2*	Pvu10G016960	176	19.74	6.73	-0.325	Nucleus, Cytoplasm
*PvuFT3*	Pvu06G009730	174	19.61	7.79	-0.296	Nucleus, Cytoplasm
*PvuFT4*	Pvu05G010880	174	19.58	6.96	-0.211	Nucleus, Cytoplasm
*PvuFT5*	Pvu05G010890	178	20.15	7.80	-0.308	Nucleus, Cytoplasm
*PvuFT6*	Pvu06G009720	178	20.01	6.51	-0.378	Nucleus, Cytoplasm
*PvuBFT1*	Pvu13G010420	173	19.44	8.45	-0.234	Nucleus, Cytoplasm
*PvuBFT2*	Pvu09G014450	173	19.58	7.76	-0.223	Nucleus, Cytoplasm
*PvuATC1*	Pvu01G034510	172	19.39	9.12	-0.276	Nucleus, Cytoplasm
*PvuATC2*	Pvu04G029210	176	19.55	8.99	-0.168	Nucleus, Cytoplasm
*PvuTFL1A*	Pvu08G001710	176	19.82	5.91	-0.309	Nucleus, Cytoplasm
*PvuTFL1B*	Pvu10G009150	226	25.10	6.75	-0.135	Nucleus, Cytoplasm
*PvuTFL1C*	Pvu07G023830	175	19.66	9.39	-0.368	Nucleus, Cytoplasm
*PvuMFT*	Pvu05G013590	179	19.47	9.60	-0.174	Nucleus, Cytoplasm
*PvuSMFT*	Pvu07G025990	168	18.29	5.44	-0.310	Nucleus, Cytoplasm

AA, amino acid; MW, molecular weight; Pi, isoelectric point; GRAVY, grand average of hydropathicity.

Gene collinearity analysis uncovered extensive events among the PEBP gene family. Specifically, 11 segmental duplication events were detected, involving the following gene pairs: *PvuFT1*/*PvuFT2*, *PvuFT4*/*PvuFT6*, *PvuACT1*/*PvuACT2*, *PvuATC1*/*PvuTFL1C*, *PvuATC1*/*PvuTFL1B*, *PvuATC2*/*PvuTFL1C*, *PvuATC2*/*PvuTFL1B*, *PvuTFL1C*/*PvuTFL1A*, *PvuTFL1C*/*PvuTFL1B*, *PvuTFL1A*/*PvuTFL1B*, *PvuBFT2*/*PvuBFT1*. In addition, two tandem duplication events were identified, involving the pairs *PvuFT3*/*PvuFT6* and *PvuFT4*/*PvuFT5* (**Figure 2B**). Collectively, these findings indicate that gene duplication, particularly segmental duplication, constitutes a predominant mechanism driving the expansion of the PEBP gene family in *P. vulgaris*.

### Gene structures and conserved motifs of PEBP proteins in *P. vulgaris*

To gain further insight into the structural features of the *PvuPEBP* genes family, we examined their exon-intron organization. As illustrated in **Figure 3B**, the intron number among *PvuPEBP* genes varies from one to five. Within the *TFL* subfamily, *PvuTFL1B* possesses the highest intron count (five), whereas *PvuSMFT*, a member of the *SMFT* subfamily, contains the lowest (one). Notably, other subfamily members share identical exon-intron configurations, suggesting a potential association between gene structure and subfamily-specific functions, regulatory pathways, or evolutionary trajectories (**Figure 3B**). To characterize the conserved protein motifs, we employed the MEME Suite to predict motif composition across the PvuPEBP family. As showed in **Figure 3C**, each PvuPEBP protein contains five to six conserved motifs. Proteins clustering within the same phylogenetic branch exhibits similar motif arrangements. All members possess Motif 3, and the majority also harbor Motif 1, 2, 4, and 5, indicating a high degree of sequence conservation among PvuPEBP.

**Figure 3:**
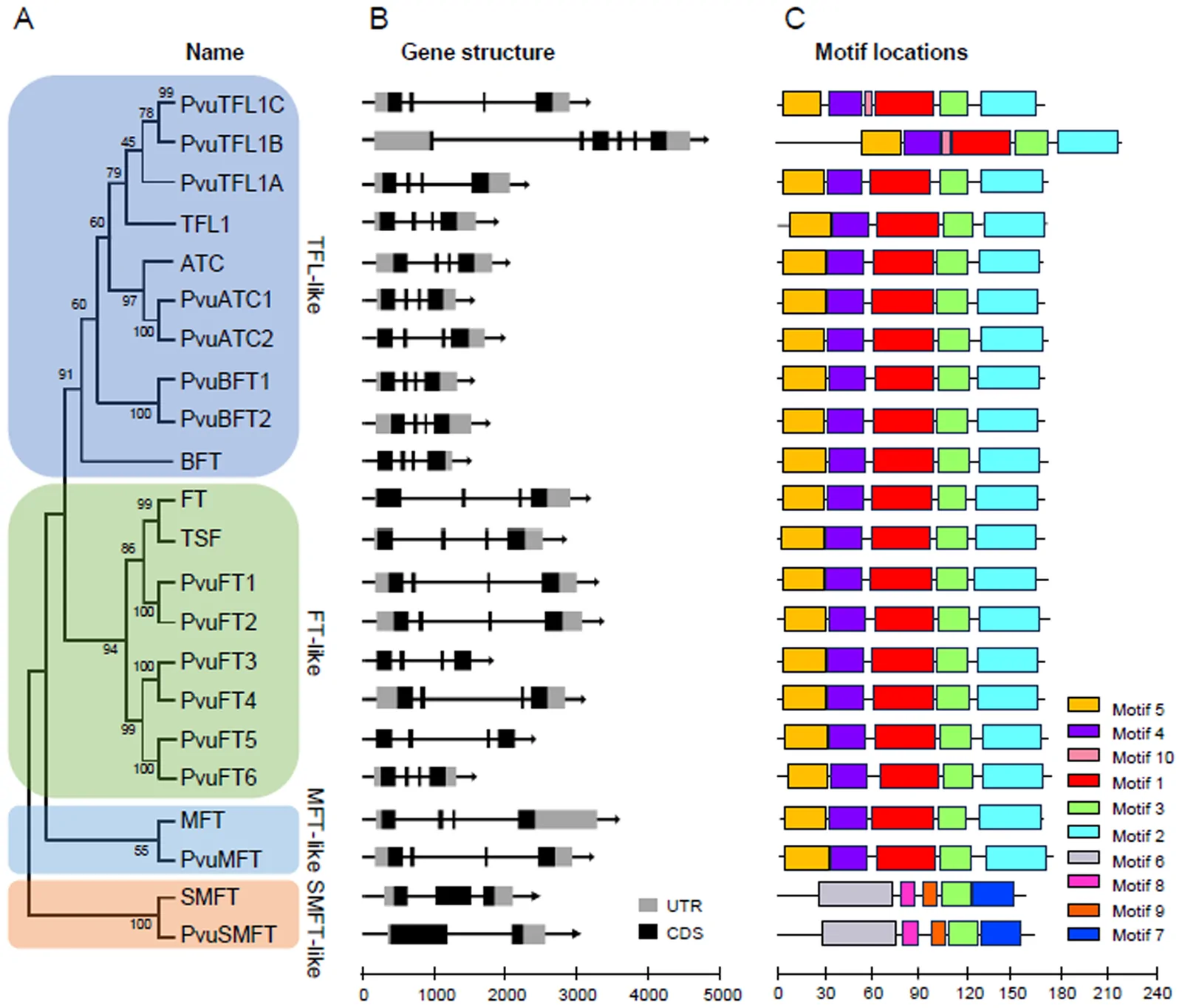
Phylogenetic relationships and structural characteristics of the PEBP family in *P. vulgaris*. **(A)** Phylogenetic tree of PEBP proteins in *P. vulgaris*, constructed using the Neighbor-Joining (NJ) method implemented in MEGA 10 software. **(B)** Gene structures of PEBP family members in *P. vulgaris*. Gray and dark boxes represent untranslated regions (UTRs) and coding sequences (CDSs), respectively; the intervening lines denote introns. The scale at the bottom indicates relative gene lengths. **(C)** Predicted motif distribution within the PEBP proteins of *P. vulgaris*. The scale at the bottom indicates relative protein lengths.

### Subcellular localization and expression patterns of *FT*-like genes in *P. vulgaris*

To investigate the potential functions of *FT*-like genes in *P. vulgaris*, we cloned the full-length coding sequences (CDSs) of *PvuFT1* through *PvuFT6*, along with *AtFT*, and fused each to the coding sequence of yellow fluorescence protein (YFP) under the control of the CaMV *35S* promoter, generating the fusion constructs *PvuFT1-YFP*, *PvuFT2-YFP*, *PvuFT3-YFP*, *PvuFT4-YFP*, *PvuFT5-YFP*, *PvuFT6-YFP*, and *AtFT-YFP* (**Figure 4A**). Transient expression assays in *Nicotiana benthamiana* leaves revealed that all YFP-tagged proteins, like the YFP-only control, exhibited fluorescence signals in both the cytoplasmic and nuclear compartments (**Figure 4B**), a pattern consistent with previous observation in other species (Zhang et al., 2023; Liu et al., 2022).

**Figure 4:**
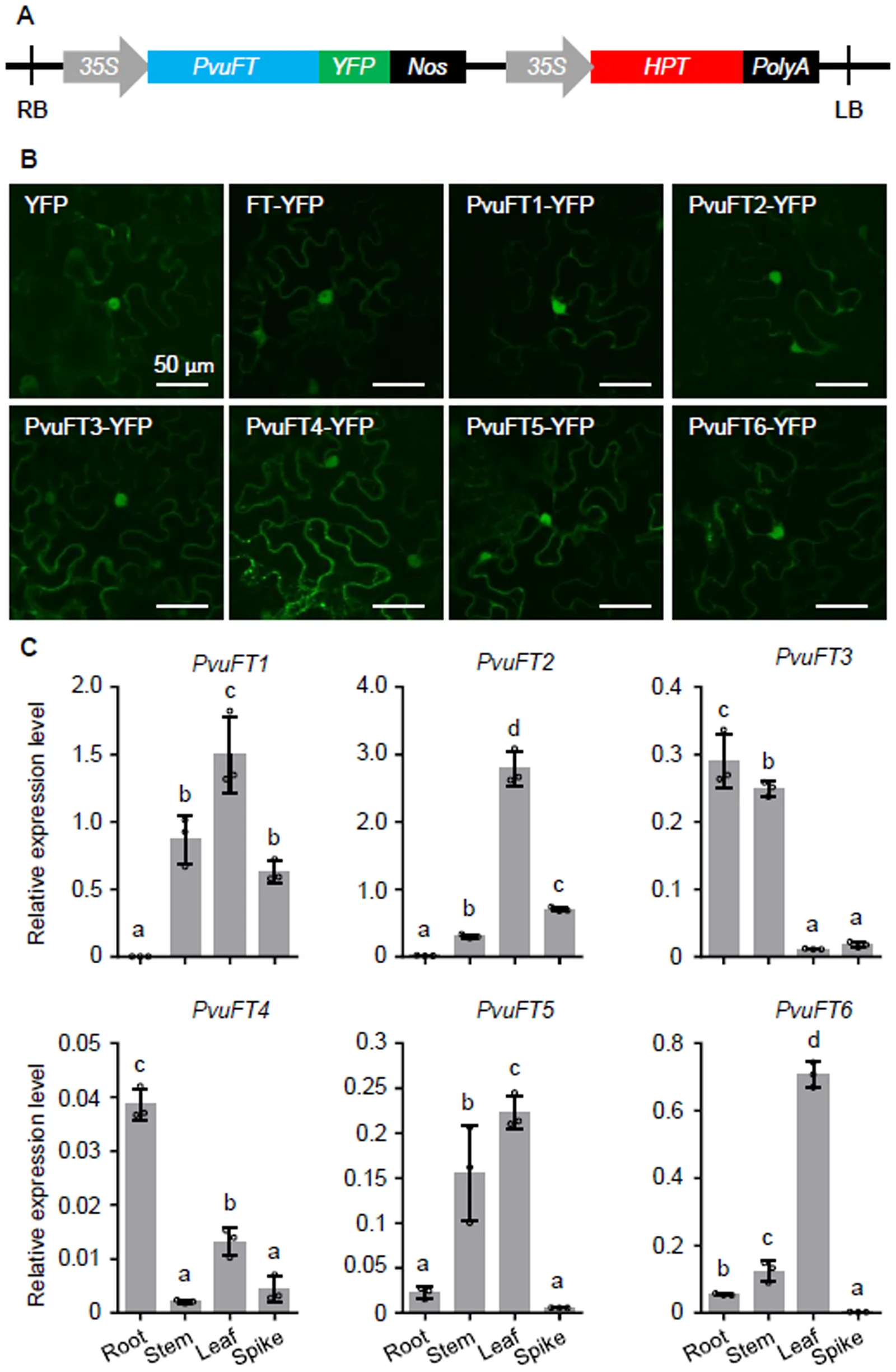
Subcellular localization and tissue-specific expression profiles of *FT*-like genes in *P. vulgaris*. **(A)** Schematic representation of the *p35S::N1-YFP* constructs, comprising *p35S::YFP*, *p35S::AtFT-YFP*, *p35S::PvuFT1-YFP*, *p35S::PvuFT2-YFP*, *p35S::PvuFT3-YFP*, *p35S::PvuFT4-YFP*, *p35S::PvuFT5-YFP*, *p35S::PvuFT6-YFP*. **(B)** Subcellular localization of YFP, AtFT-YFP, and PvuFT1-YFP through PvuFT6-YFP fusion proteins. Scale bars = 50 μm. **(C)** Relative expression levels of *FT*-like genes across various organs of *P. vulgaris*. The x-axis indicates the examined tissues, and the y-axis denotes transcript abundance normalized to the reference gene *Pvu06G020000*. Values represent the mean ± SD. Different lowercase letters indicate statistically significant differences (*P* < 0.05, one-way ANOVA with Tukey’s *post-hoc* test).

To examine the spatial expression profiles of these *FT*-like genes, we performed quantitative RT-PCR (qRT-PCR) to measure their transcript levels in buds, stems, leaves, and roots. As shown in **Figure 4C**, the six *PvuFT* genes displayed distinct tissue-specific expression patterns. *PvuFT1* and *PvuFT2* were most abundantly expressed in leaves, with moderate levels in stems and flower buds and undetectable expression in roots. *PvuFT5* and *PvuFT6* also exhibited predominant expression in leaves, followed by stems and roots, but showed barely detectable transcript accumulation in flower buds. In contrast, *PvuFT3* and *PvuFT4* were expressed across all four tissues examined, with the highest transcript abundance detected in roots. Collectively, the marked differences in both expression profiles and transcript levels among the six *PvuFT* genes suggest that they may undergone functional divergence and play distinct biological roles in *P. vulgaris*.

### Over-expression of *PvuFT1* and *PvuFT2* in *Arabidopsis* strongly accelerated flowering

To determine whether the *PvuFT*-like genes share conserved functions in flowering-time regulation, we introduced *PvuFT1-YFP* through *PvuFT6-YFP*, along with the positive control *AtFT-YFP*, into *Arabidopsis* (**Figure 4A**). Following hygromycin resistance selection and PCR-based genotyping, T_2_ transgenic lines were selected for further phenotypic analysis based on their transgene expression levels (**Figure 5B**). Under inductive long-day photoperiod conditions, transgenic plants overexpressing *PvuFT1* and *PvuFT2* exhibited early flowering and produced significantly fewer rosette leaves at the time of floral transition, phenotypes analogous to those observed in the positive control *AtFT-YFP* lines (**Table 2**; **Figures 5A, C, D**). By contrast, while *PvuFT5* overexpression resulted in a modest late-flowering phenotype, transgenic lines carrying *PvuFT3*, *PvuFT4*, and *PvuFT6* did not show any discernible alteration in flowering time relative to the controls (**Table 2**). Collectively, these results demonstrate that *PvuFT1* and *PvuFT2* are structural and functional homologs of *AtFT* in *P. vulgaris* and are capable of exerting the floral-promoting activity of the endogenous florigen.

**Figure 5:**
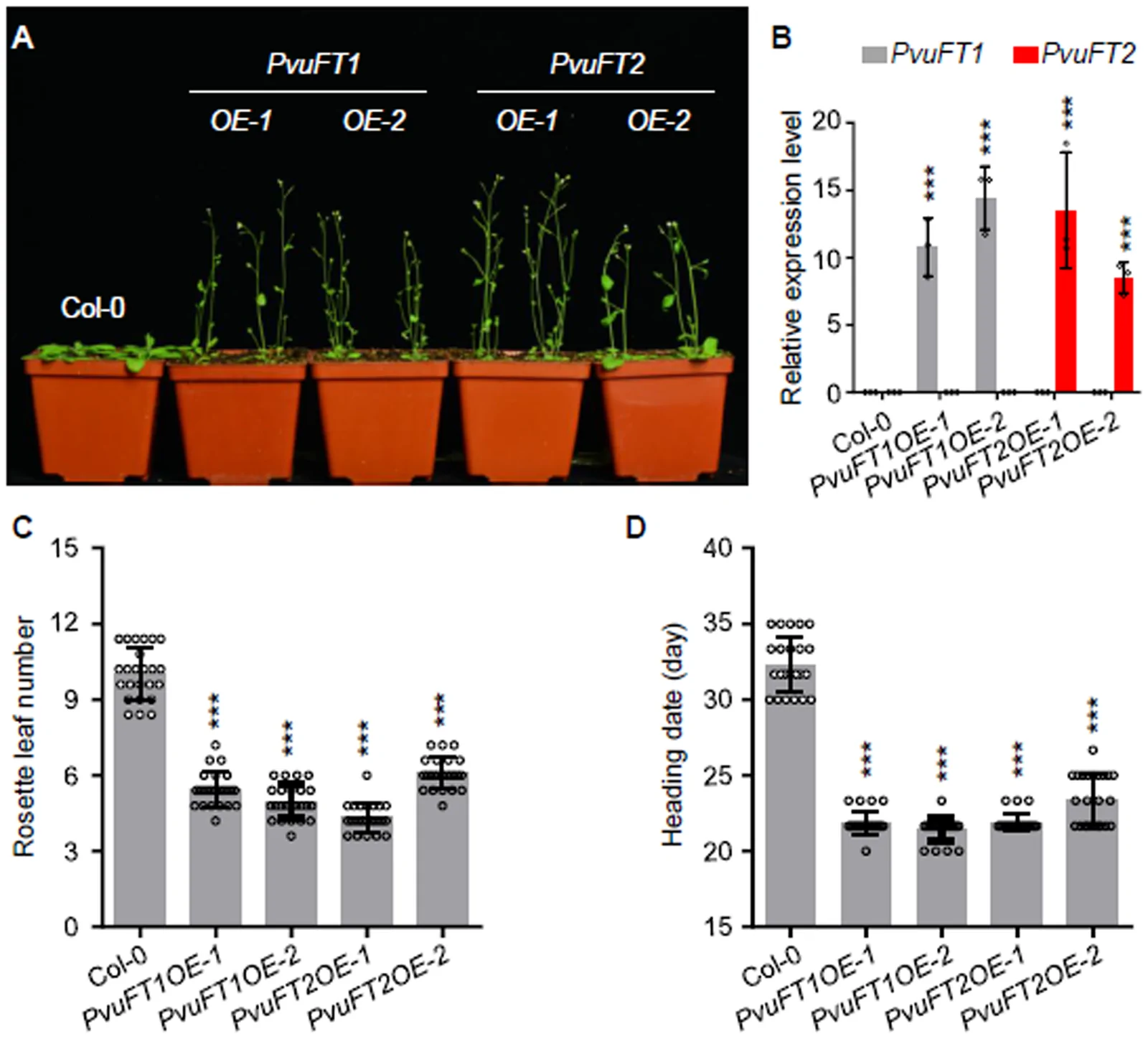
The function of *PvuFT1* and *PvuFT2* in Arabidopsis. **(A)** The phenotype of Arabidopsis with the overexpression of *PvuFT1* and *PvuFT2* genes. All plants were grown under long-day conditions with 16 h of light and 8 h of darkness at 22 °C. **(B)** The relative expression level of *PvuFT1* and *PvuFT2* in transgenic Arabidopsis. **(C)** The number of rosette leaves. **(D)** The flowering days. Values represent the mean ± SD. The asterisk denotes a significantly different compared Col-0 (***P < 0.001, based on T-Test).

**Table 2:** The rosette leaf number and heading date were evaluated in Arabidopsis plants ectopically overexpressing *PvuFT*-like genes.

Genotype	Rosette leaf number	Heading date
Col-0	10.00 + 1.03	31.11 + 1.08
*FTOE-1*	3.50 + 1.29***	22.21 + 1.01***
*FTOE-3*	3.83 + 0.62***	22.68 + 2.31***
*FTOE-4*	3.67 + 1.15***	22.90 + 2.14***
*PvuFT3OE-1*	10.51 + 1.02	31.23 + 2.23
*PvuFT3OE-2*	10.57 + 1.62	31.30 + 3.50
*PvuFT3OE-3*	10.33 + 1.12	31.00 + 5.13
*PvuFT4OE-1*	10.20 + 1.21	31.22 + 5.19
*PvuFT4OE-2*	10.09 + 1.22	31.75 + 1.48
*PvuFT4OE-3*	10.83 + 1.94	31.17 + 1.94
*PvuFT5OE-1*	20.75 + 3.20***	44.20 + 1.10***
*PvuFT5OE-2*	20.00 + 8.12***	42.40 + 4.34***
*PvuFT5OE-3*	20.83 + 4.26***	40.75 + 2.36***
*PvuFT6OE-1*	9.92 + 2.43	31.44 + 1.59
*PvuFT6OE-2*	9.57 + 1.79	31.67 + 1.80
*PvuFT6OE-3*	9.60 + 1.43	31.30 + 2.21
*PvuFT6OE-4*	9.88 + 0.81	31.56 + 1.50
*PvuFT6OE-5*	10.06 + 1.00	30.25 + 0.45

Values represent the mean ± SD. Asterisks indicate statistically significant differences based on Student’s t-test (***P < 0.001).

### *PvuFT1* and *PvuFT2* accelerate flowering in *P. vulgaris*

To investigate whether the *PvuFT1* and *PvuFT2* are functionally in flowering-time regulation in *P. vulgaris*, we first compared the flowering time of two representative accessions, Yiyang and Guiyang, which exhibit contrasting flowering times (**Figure 1**). Under long-day conditions, Guiyang displayed a significant earlier flowering phenotype than Yiyang (**Figures 6A, B**). We next examined the temporal expression profiles of *PvuFT1* and *PvuFT2* in leaves of both accessions. From weeks 5 to 8 post-planting, transcript levels of both genes remained low and comparable between the two accessions. However, starting at week 9, *PvuFT1* and *PvuFT2* transcript abundance increased markedly in Guiyang, whereas no such elevation was observed in Yiyang (**Figure 6C**). This expression pattern is consistent with the earlier flowering phenotype of Guiyang (**Figures 1B, C**, **6A, B**), suggesting that *PvuFT1* and *PvuFT2* may contribute to the regulation of flowering time in *P. vulgaris*.

**Figure 6:**
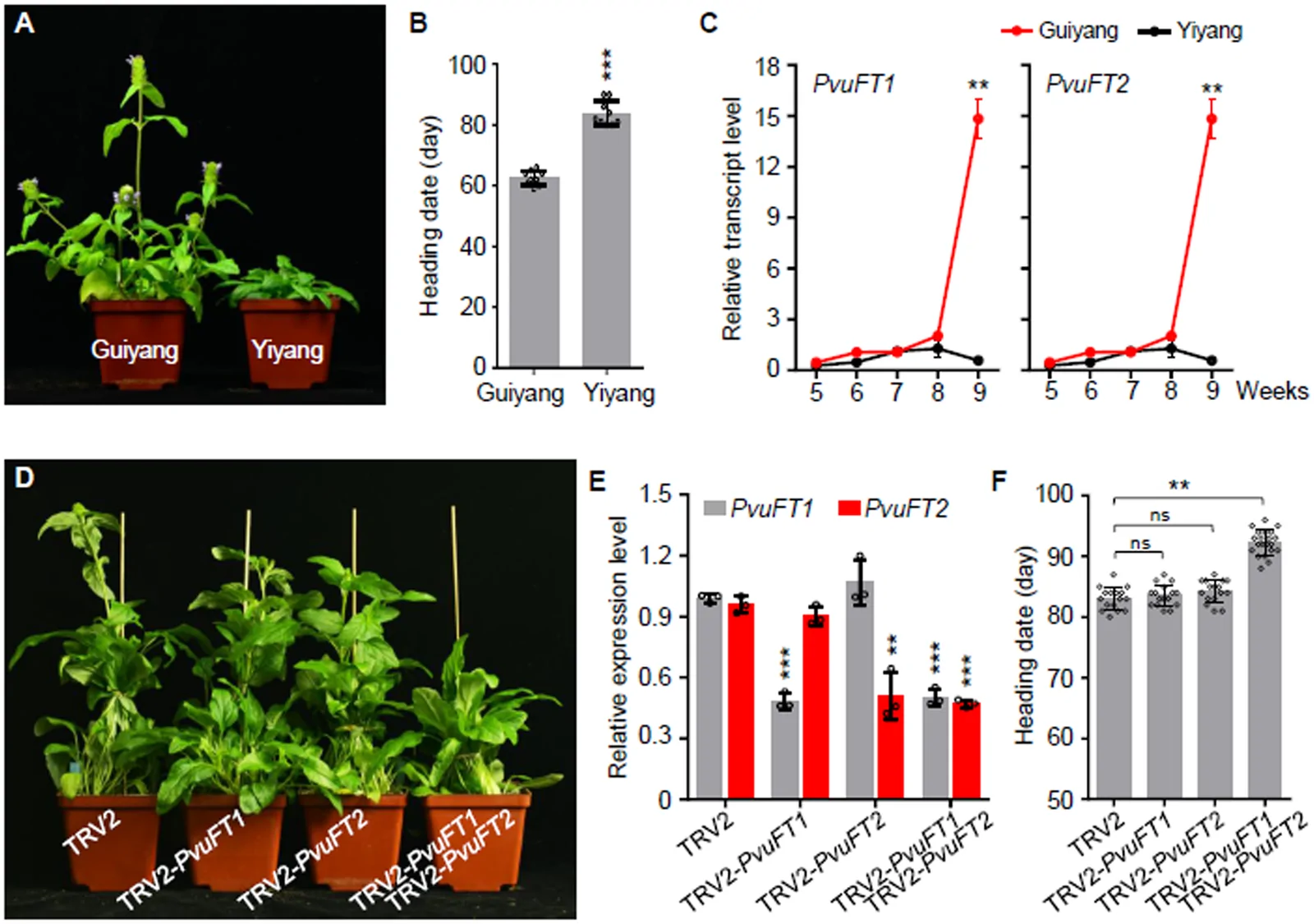
Roles of *PvuFT1* and *PvuFT2* in flowering time regulation in *P. vulgaris.* **(A)** Phenotypic comparison of *P. vulgaris* populations originating from Guiyang and Yiyang. **(B)** Flowering time of the two populations. **(C)** Relative transcript levels of *PvuFT1* and *PvuFT2* in the two populations. **(D)** Representative phenotypes of plants subjected to TRV2 (empty vector control), TRV2-*PvuFT1*, TRV2-*PvuFT2*, and combined TRV2-*PvuFT1* TRV2-*PvuFT2* treatments. **(E)** Relative transcript levels of *PvuFT1* and *PvuFT2* in the respective virus-induced gene silencing (VIGS) lines. **(F)** Flowering time of the VIGS lines. All plants were grown under long-day conditions (16 h light/8 h dark) at 22 °C. Values represent the mean ± SD. Asterisks indicate statistically significant differences based on Student’s t-test (**P < 0.01, ***P < 0.001).

To further validate the floral-promoting roles of *PvuFT1* and *PvuFT2*, we employed a virus-induced gene silencing (VIGS) system to suppress their expression in the leaves of the Yiyang accession. Quantitative RT-PCR analysis revealed that plants infiltrated with TRV2-*PvuFT1* or TRV2-*PvuFT2* alone retained approximately 50% of the corresponding transcript levels relative to the TRV2 control, while plants co-infiltrated with both TRV2-*PvuFT1* and TRV2-*PvuFT2* exhibited a more pronounced silencing phenotype, with transcript levels of both genes reduced to approximately 50% simultaneously (**Figures 6D, E**). Phenotypic analysis of flowering time showed that single-gene silencing lines flowered normally compared with the TRV2 control, whereas double-silenced plants exhibited a significant delay in flowering (**Figure 6F**). Collectively, these results demonstrate that both *PvuFT1* and *PvuFT2* promote flowering in *P. vulgaris*. Together, our findings establish that these two *FT*-like genes within the PEBP family serve as key determinants of flowering-time variation in *P. vulgaris.*

## Discussion

*P. vulgaris* possesses significant medicinal and industrial value. In this study, we characterized the PEBP gene family in *P. vulgaris* and demonstrated that *PvuFT1* and *PvuFT2* play pivotal roles in flowering time regulation. Our data suggest that gene duplication drove functional divergence among FT-like paralogs, with two homologs promoting floral transition. These findings address a critical knowledge gap in the regulation of flowering time in *P. vulgaris* and establish a direct functional link between *FT* homologs and reproductive growth—the developmental stage that determines the yield of medicinal tissues.

*FT*, a member of the PEBP family, functions as a central integrator of flowering signals and plays a pivotal role in the floral transition (Corbesier et al., 2007). Through genome-wide identification, we recovered 15 PEBP family members in *P. vulgaris*, including six *FT*-like genes (*PvuFT1* through *PvuFT6*). Subcellular localization assays revealed that all six FT-like proteins are targeted to both the nucleus and the peripheral cytoplasm. Expression profiling demonstrated that *PvuFT1* and *PvuFT2* are predominantly and highly expressed in leaves, implicating their potential involvement in the floral inductive process. Notably, in addition to their leaf-specific enrichment, both genes also exhibited detectable transcript accumulation in flower buds, consistent with recent reports that *FT* is also expressed in floral primordia and contributes to flowering promotion in *Arabidopsis* (Gao et al., 2025a). Overexpression of the six *PvuFT* genes in *Arabidopsis* further confirmed that only *PvuFT1* and *PvuFT2* possess the capacity to promote flowering (**Figure 5**). Moreover, the early-flowering Guiyang accession displayed significantly higher transcript levels of *PvuFT1* and *PvuFT2* compared with the late-flowering Yiyang accession (**Figures 6A–C**). Conversely, VIGS-mediated downregulation of *PvuFT1* and *PvuFT2* in the Yiyang background markedly delayed flowering, further corroborating their floral-promoting functions (**Figures 6D–F**). In angiosperms, the florigen gene *FT* generally promotes flowering, whereas certain *FT* homologs have been reported to repress it (Wickland and Hanzawa, 2015; Huang et al., 2024). For instance, in sugar beet (*Beta vulgaris*), two FT homologs exhibit opposing functions, a difference attributed to two amino acid substitutions in the segment B region encoded by the fourth exon (Pin et al., 2010). In *P. vulgaris*, we identified six *PvuFT*-like genes. Among these, *PvuFT1* and *PvuFT2* function as flowering promoters, while overexpression of *PvuFT5* in *Arabidopsis thaliana* resulted in flowering repression (**Table 2**). The coexistence of both promoter-type (*PvuFT1/2*) and repressor-type (*PvuFT5*) *FT*-like genes in *P. vulgaris* suggests a finely tuned regulatory network that may modulate flowering time in response to environmental cues. This may represent an adaptive strategy in a perennial medicinal plant, where optimal flowering timing is crucial for both reproductive success and secondary metabolite accumulation.

Plants exhibit adaptive phenological adaptations to synchronize flowering with prevailing environmental conditions, thereby ensuring successful reproductive completion. Local populations often undergo regional selection, which favors genetic variants that confer adaptive modifications in morphological and developmental traits (Gao et al., 2025b). In this study, we observed significant inter-accession variation in flowering time when *P. vulgaris* ecotypes originating from Guiyang, Liangping, Queshan, and Yiyang were cultivated under uniform field conditions at Shanghai Chenshan Botanical Garden. Given that all accessions were grown in a common environment, the observed divergence in flowering time strongly suggests the presence of underlying genetic variation among these populations. Appropriate flowering time is critical for plant environmental adaptation, and *FT*, a quantitative trait locus, plays a pivotal role in flowering time regulation (Schwartz et al., 2009; Liu et al., 2014). Consistent with this functional importance, our study revealed that in the ninth week, *PvuFT* transcript levels were significantly higher in the early-flowering Guiyang accession of *P. vulgaris* than in the late-flowering Yiyang accession (**Figure 6C**). Sequence analysis showed that the amino acid sequences of PvuFT1 and PvuFT2 are identical between the two accessions; however, their promoter regions exhibit nucleotide polymorphisms (data not shown). We will further investigate whether these promoter sequence variations function as cis-regulatory elements that directly modulate the transcription of *PvuFT1* and *PvuFT2*, or whether other trans—acting factors are involved in regulating this process.

In summary, this study discovered that *P. vulgaris* germplasm from four geographic regions exhibited differential flowering times. Systematic characterization of the PEBP gene family identified 15 members, including six FT-like proteins, all of which localized to both the nucleus and cytoplasm. These proteins displayed tissue-specific expression profiles across roots, stems, leaves, and buds. Overexpression of *PvuFT1* and *PvuFT2* significantly accelerated flowering in *Arabidopsis thaliana*, while their concurrent silencing via VIGS markedly delayed flowering in *P. vulgaris*. Collectively, these findings confirm *PvuFT1* and *PvuFT2* as critical regulators of flowering time and provide a molecular basis for breeding elite varieties with optimized growth durations, offering a molecular foundation for breeding *P. vulgaris* cultivars with tailored growth cycles to meet diverse production requirements. The precise control of flowering time is a central question in developmental biology, and the FT-centered regulatory network is known to integrate both endogenous developmental signals and external environmental signals. Although this framework has been extensively studied in model species, our understanding in non-model plants—especially wild relatives—remains limited. Wild plants, having long adapted to heterogeneous natural habitats, harbor rich natural genetic variation that likely represents molecular footprints of natural selection on loci governing flowering plasticity. Thus, future endeavors that combine multi-omics approaches with gene editing technologies to systematically dissect the flowering regulatory machinery in wild plants will not only deepen our evolutionary insights but also facilitate the conservation and sustainable use of valuable genetic resources. This line of research therefore represents a direction for the field.

## Data Availability

The datasets presented in this study can be found in online repositories. The names of the repository/repositories and accession number(s) can be found in the article/**Supplementary Material**.
